# Central Texas Medical Society

**Published:** 1899-08

**Authors:** N. A. Olive

**Affiliations:** Secretary


### Society Notes.

### Central Texas Medical Society.

At the regular meeting held at Waco, Texas, July 11th and 12th ult., there was a good attendance and an interesting program was carried out. The JOURNAL is indebted to the secretary, Dr. N. A. Olive, for a full report of the meeting, together with the discussions on the papers read, and we give below a summary of same.

Vice-President J. C. J. King, of Waco, in the chair. First paper was by Dr. A. P. Brown, of Fort Worth, read by the secretary, Dr. Brown not being present. It was entitled "Facts," and consisted of a statement of some of the doctor's experiences in a long professional life. He says he has treated diphtheria since 1858 and never lost a case. It is to be regretted that the doctor did not give his method of treatment. The doctor says also that he never had to operate for appendicitis, and expresses the opinion that operation is never necessary "except in chronic recurrent cases." One of his "facts" which he states in the paper it that "vinegar is a quick antidote to carbolic acid." The doctor has found absorbent cotton saturated with lemon juice to be the best remedy in nose bleed. He gives the following formula for a corn salve, which he has found very efficacious:

℞ Acid salicylic.......................................ʒj

Vaseline.......................................℥ss

Sig. Apply to the corn three nights, confining its action to the corn, as if allowed to come in contact with the skin it will excoriate.

For soft corn he recommends:

℞ Fl. ext. cannabis ind.......................................ʒj

Acid salicylic.......................................ʒj

Sulph. ether, q. s. ad.......................................℥j

Dr. Brown's paper was discussed by Drs. Sears, Wallace (D. R.), Curtis, Park *et al.* Dr. Sears said: "Dr. Brown says he has treated diphtheria since 1858 and never lost a patient. He must have had very little diphtheria. I've seen tonsilitis that might have been called diphtheria. If a man says he has treated four hundred cases of diphtheria and never lost one I think he is—prevaricating." Dr. D. R. Wallace had "met a man who said he had treated lots of double pneumonia and never lost a case." "I told him," says the doctor, "that he had never treated many cases, and," he added, "I consider it so with Dr. Brown: he never had much diphtheria." Dr. Curtis said that Dr. Brown lived in a windy city. This fact would account for the paper, (or something to that effect).

Dr. R. C. Fisher read a paper on "constipation," which was well received and was discussed at length. Dr. Grace, of Walnut Springs, said: "In the discussion of this valuable paper there is one point of great interest that has not yet been brought out. I refer to the serious consequences of continued constipation. When the bowel ceases to properly perform its function the other excretory organs, especially the skin and kidneys take up and excrete the solids to a more or less extent, thereby overtaxing their physiological strength. We know that beer drinkers are often sufferers from Bright's disease. The beer producing copious passages of urine of low specific gravity. Then if this will irritate the little tubules of the kidney and bring about a chronic nephritis, how much more likely is the urine of a constipated subject, with its irritating high specific gravity and increased amounts of solids, to bring about this dread disease. This, with other serious consequences, should make this a very interesting subject to the profession."

Dr. S. E. Milliken, of Dallas, gave a demonstration of an improved operation for hernia, which, the secretary says, was one of the most attractive features of the meeting. Dr. Milliken uses the silver suture of Dr. Harris, of Chicago.

Dr. Riley spoke ex tempore on "Laryngeal Tuberculosis," in default of paper. The presentation of the case by Dr. Riley was discussed by most of the members present. Dr. J. M. Woodson, of Temple, chairman of section on ophthalmology, etc., read a paper on "Sympathetic Ophthalmia." We will publish a synopsis of this paper in next issue, together with the discussion.

A paper by Dr. J. V. Shoemaker, of Philadelphia, on the Therapeutic Value of Cannabis Indica was read and discussed. The paper was not sent us; hence we omit the discussion as reported by the secretary. Dr. W. A. Howard read a paper on "Ingrowing Nail." He cauterizes with nitrate silver and inserts oil paper or tin foil under the nail. The general sense of the meeting, as developed in discussion, was in favor of an operation, however.

At the evening session Hon. John G. Winter, of Waco, delivered an address on "The Lawyer and the Doctor." We publish this paper herewith and call attention to it. It "created a rather lively discussion by both lawyers and doctors," says the secretary. A vote of thanks was given Col. Winter, and the paper was ordered to be filed with the archives of the society and a copy sent to "a leading medical journal for publication."

### SECOND DAY.

Meeting called to order at 10:30 a. m.

Vice-President J. C. J. King in the chair.

Members present, Drs. J. H. Sears, J. C. J. King, G. B. Fosuce, H. C. Ghent, M. B. Grace, A. M. Curtis, J. J. Dean, M. M. Smith, S. M. Jenkins, J. M. Hale, H. C. Black, J. T. Harrington, T. C. Walker and N. A. Olive.

First subject,

### "THE MOSQUITO THEORY OF MALARIAL FEVER."

Paper by Dr. J. H. Sears, Waco.

### DISCUSSION.

Dr. H. C. Ghent: I am glad the doctor read his paper. My observation is when we have an overflow, something produces sickness. I do not know what it is. We have various types of malarial fever, chills, intermittent fever, remittent fever, congestive fever, black jaundice, etc., following a flood. The best prophylactic is quinine.

Look out this year for bilious fevers. Quinine for treatment is good and as a prophylactic it is a success.

Dr. Grace: Three elements are required to produce malaria, viz.: heat, moisture and vegetation. We should employ all possible prophylactic measures. I regard quinine as the panacea in malaria. I treat with quinine by beginning with the remission and give five grains every two hours until thirty to forty grains are given. In from two to four days, I can abort those cases susceptible of abortion in this manner.

Quinine given in excess will destroy the coagulating quality of the blood, thus producing hemorrhage. Too much is given; it is often abused.

Dr. J. T. Harrington: I find some patients more susceptible to the drug than others. You must give more quinine to one than another can take. I give it combined with arsenious acid and antifebrine or phenacetine. This is more efficient than quinine alone. The coal tar products prevent nervousness to some extent.

Dr. M. M. Smith (of Austin): I would like to hear the experience of some of the older members of the society as to the prophylactic effect of coffee, taken before going out in the morning. I think it has good effect. There is no doubt as to the value of quinine in eliminating malaria from the system. But we must attend to the bowels and kidneys.

Dr. Curtis: Dr. Sears thinks mosquitoes cause malaria. I think if a person will drink boiled water, mosquitoes may roost on him and he will not have chills. If you give quinine at all times and under all circumstances, you are liable to have haematuria and other malignant forms of malaria. You may give quinine daily and have chills for years. Arouse your secretions properly and you will cure your trouble. If you give quinine, use it in an acid solution for its best effect. If you persist in giving coal tar preparations you will sooner or later bid your patient good bye. Arouse your secretions properly, then you can give quinine with better effect and greater safety.

Dr. Hill: I have had patients drink boiled water altogether and they had chills same as other people.

Dr. H. C. Ghent: I do not advocate quinine as a treatment, but as a prophylactic. I was not discussing the treatment.

Dr. H. C. Black: I suppose every one recognizes the value of calomel in malaria. Dr. Joseph Jones, of New Orleans, said if he was to use one drug only for malaria, he would use calomel. As a prophylactic quinine is the chief. In treatment quinine has often failed of result until calomel has been used. Boiled water is a useful preventive measure. We should not lose sight of the action of the skin. Bathe and keep clean the greatest eliminating organ of the body. Warburg's tincture given in large doses after the action of the calomel will keep up the secretions and keep tongue and skin moist. I never attached much value to arsenic in malaria. As a prophylactic it is good. I give it to those who do not bear quinine.

Dr. J. C. J. King: I do not think overflows always bring sickness. It was exactly the reverse here one year. You can not count on sickness following every overflow.

Dr. Ghent: I think some are discussing prophylaxis and others treatment. No man would be great enough fool to treat malarial fever with quinine alone. I give calomel, Dover's powder and other drugs.

Dr. H. C. Black: I nearly always give quinine and calomel together as a prophylactic. Think I have kept off many attacks by this means alone. Calomel is as important in prophylaxis as is quinine. For enlarged spleen they used to give one grain of iodoform, in capsule, three times daily, in the New Orleans hospital, with the best results. I have not used it here, but think it would act equally as well as there. The spleen is greatly benefited by its use.

Dr. N. A. Olive: I am surprised to hear any one speak as if he doubted the value of quinine given in proper dosage, under proper conditions and at the right time. My experience is that with its skillful administration you can almost always look for the happiest results. The fact that quinine is indicated does not signify that other good remedies are contra-indicated. The fact that quinine does not fill the bill of calomel and all other remedies that may at time be demanded, is no argument against the efficacy of quinine. I give it fearlessly when in my judgment it is demanded. Any remedy that we use daily is capable of doing harm in careless and unskilled hands, but we do not rise to denounce them for that reason. 'Tis true a chill habit may be present and you may go on having chills for years in spite of quinine and other good antiperiodics, but this is no argument against their efficacy. One can establish a certain amount of tolerance to quinine as with a habit of any kind and it will not have its usual physiological effect. I think one may have chills as a matter of habit after all malaria is eliminated from the system, the patient has grown accustomed to it, the nervous system is prepared for the paroxysm and it comes on as of yore. These cases may be arrested by a shock as in dashing a bucket of ice water on the patient at onset of the attack. They often will never return when treated in this manner. If a shock to the nervous system is all that is required to cure, then certainly quinine is not indicated, is not admissible and will not cure. Another point about quinine is that it sometimes acts better in combination with other anti-periodics, than alone. Arsenic ofttimes seems to be a clincher for quinine. A good combination for chronic malarial toxæmia is:

| | | |
|---|---|---|
| ℞ | Strych. sulph | gr. $\frac{1}{30}$ |
| | Acid arsen | gr. $\frac{1}{20}$ |
| | Piperin | gr. j |
| | Quinia sulph | ʒj |
| | M. ft. cap. no | j |

Sig. Give one capsule every four hours during the day.

Quinine is the chief ingredient in all patent nostrums that have acquired any reputation for the relief of malaria in any form. In my opinion it is more nearly a specific for malaria than any remedy for almost any known disease. If it is given intelligently it can not be given amiss.

Dr. Sears: The discussion has gone over the ground very well. The blood of the malarial subject is full of the plasmodium, and every chill destroys forty per cent. of the hæmoglobin of the red blood corpuscles that contain the plasmodium. Give quinine in proper form and sufficient quantity to get into the blood and destroy the plasmodium. Twenty grains may do the work in the incipiency; but with every additional paroxysm you will have to give more quinine. Dr. Grace should have given ten to twenty-five grains of quinine every three hours until the fever was broken, then five grains three times daily.

Do not quit giving medicine because the fever has stopped. When liver and spleen are enlarged give quinine in twenty grain doses every three hours until they disappear under the ribs ("or under the soil," said a voice from the audience). You would not have so much abscess of liver if quinine was given in that manner. Calomel is not good in enlarged spleen. Biliousness is often caused from eating some things in excessive quantity or improper form. In that case castor oil would act as well as anything. Anything that will depress the system during the presence of the plasmodium malaria may bring on a paroxysm.

Eliminate the malaria through the skin and kidneys, and especially eliminate through the grandest of all sewer systems of man—

the bowels. Meet the indications as they arise. Every fellow has his own idiosyncrasies. If Dr. Grace had given some form of opium with his quinine he would have gotten better results. A large dose of quinine will put a patient to sleep when a small one would make him nervous. The fluid extract of common thistle will reduce the size of the spleen. There are no better hæmatics than iron, quinine, strychnia and arsenic. Arsenic stimulates the capillary circulation. Quinine and strychnine are good tonics. As to coffee, it is always well to eat before going out. Do not go out before the sun rises. If I knew of nothing better to give as a remedy for malaria, I'd do like Dr. Curtis, I'd give boiled water. It is better to do this than give remedies and do harm.

I would not give quinine in black jaundice, as Dr. King did over here a few years ago, and he like to have killed his patient, too.

Dr. King: You are mistaken; I never give quinine in black jaundice. It was some other man, and you have forgotten who it was.

Dr. Sears: No, you don't give it in black jaundice since you quit.

Dr. King: I never did.

Dr. Sears: In congestive chill give twenty to forty grains of quinine and repeat every two hours until relieved. If there is hyperpyrexia and delirium give an antipyretic to cool the fever, and follow immediately with quinine.

Dr. Grace: The reports of the surgeons from Cuba are to the effect that soldiers cured with quinine have a recurrence of malaria, and those cured by other means have no return. How does Dr. Sears account for that?

Dr. Sears: I don't account for it, for I don't believe it.

Discussion closed.

Motion to publish Prof. Shoemaker's excellent paper on "Cannabis Indica" carried.

A committee, consisting of Drs. Curtis, Foscue and Grace were appointed to select a place of meeting and Chairman of Sections and reported as follows:

Time of Meeting—Second Tuesday and Wednesday in January, 1900. Place—Waco, Texas.

(1) Section of General Medicine—Dr. H. C. Ghent, Belton, Chairman.

(2) Section of Diseases of Nervous System—Dr. M. L. Graves, San Antonio, Chairman.

(3) Section of Ophthalmology and Otology—Dr. C. Guy Reily, Waco, Chairman.

(4) Section of Surgery—Dr. J. M. Frazier, Belton, Chairman.

(5) Section of Obstetrics and Gynecology—Dr. M. B. Grace, Walnut, Chairman.

Motion to adjourn to next regular meeting carried.

N. A. OLIVE, Secretary.

